# Supplementary material for: Inferring a simple mechanism for alpha-blocking by fitting a neural population model to EEG spectra
Source: PLoS Comput Biol. 2020 Apr 30;16(4):e1007662. doi: 10.1371/journal.pcbi.1007662 (PMC7217488; doi:10.1371/journal.pcbi.1007662)
Supplement: Attachment — Submitted filename: Cover letter + response to reviewers.pdf [file pcbi.1007662.s002.pdf]

Agus Hartoyo
Optical Sciences Centre, Swinburne University of Technology
Hawthorn, Victoria, Australia 3122
ahartoyo@swin.edu.au

Subject: Submission of revised manuscript PCOMPBIOL-D-20-00063 April 2, 2020

Dr. Peter Neal Taylor
Associate Editor
PLOS Computational Biology

Prof. Lyle Graham
Deputy Editor
PLOS Computational Biology

Dear Dr. Peter Neal Taylor / Prof. Lyle Graham

Thank you for your email dated March 25, 2020 and for providing us with the opportunity to resubmit a revised version of the manuscript PCOMPBIOL-D-20-00063. We would like to express our gratitude to the reviewers for their constructive feedback.

We have revised the manuscript in line with the reviewer feedback. Key modifications include:

1. Display of the model equations as per the suggestion from Reviewer#1;

2. Speculation about the source of the extra-cortical input suggested by Reviewer#1 and providing a neuroanatomical rationale as requested by Reviewer#2;

3. Physiological explanation as to why the excitatory input to inhibitory neurons (p_{ei}) is the major determinant of changes in alpha rhythm while the excitatory input to excitatory neurons (p_{ee}) is not, as per the recommendation from Reviewer#2.

Our specific responses are detailed in the table below, which lists the reviewer's comments, our response, and the corresponding change we made to the manuscript.

We look forward to hearing back from you.

Sincerely yours,

Agus Hartoyo, Peter Cadusch, David Liley, Damien Hicks

No	Suggestion, question, or comment from Reviewer #1	Authors’ response	Change in the manuscript
1.1	“The link to access the EEG data is not working. I believe the correct is: https://archive.physionet.org/pn4/eegmmidb/ . Please, check it.”	Thank you for pointing out the hyperlink error. We have made the suggested amendment.	In the revised version of the manuscript, we have changed ‘https://www.physionet.org/pn4/eegmmidb/’ to ‘https://archive.physionet.org/pn4/eegmmidb/’ as suggested, which you can see on page 3 line 73.
1.2	“Although is mentioned the neuronal population model used and well-referenced, I believe that it would be more clear and easier for the reader to understand if the authors show explicitly the equations.”	We agree and have added the equations to the main paper.	We have added the model equations to the “Neural population model” section on page 4 of the main paper.
1.3	“The source of the extra-cortical input (pei) and its limitations could be better discussed. Extra-cortical inputs include other sources than thalamus and, depending on the source, the cortical layers and the interneurons receiving it might be different. Moreover, it also depends if you are looking at a primary or higher-order area of the cortex. In that way, still there is no specific answer about who is driving the alpha-blocking. Enriching this discussion will clarify the limitations and the possible ways to test it through experiments and more detailed models.”	We agree that discussion about the source of the extra-cortical input needed to be expanded upon. As mentioned, our model does not specify the source of the extra-cortical input, nor is it detailed enough to specify the region of the cortex targeted. We suspect that explicit modeling of such details would increase the complexity of the (already difficult) parameter estimation problem. Nevertheless, because alpha-blocking appears throughout cortex, we expect the source to be thalamic rather than cortico-cortical. We also discuss why thalamic inputs are likely to affect inhibitory cortical neurons primarily.	Speculation about the thalamic source is in the paragraph beginning on line 238. Discussion about why alpha-blocking is likely due to excitation of inhibitory neurons begins on line 246.

No	Suggestion, question, or comment from Reviewer #2	Author's response	Change in the manuscript
2.1	"While compelling, the authors should more clearly explain a neuroanatomical rationale for why only inputs to the interneurons regulate alpha. Is there a thalamic source for this? Could it be provided via thalamic matrix inputs (Biological Psychiatry 87(8):770)? Authors should cite relevant literature."	Our model is restricted to the dynamics of the cortex, a limitation that we believe allows us to successfully achieve the inverse problem. Though we speculate that the source of the external input is thalamic, any further detail would be well beyond the scope of our model. We have cited literature to explain why a thalamic source would favor inhibition.	Citations describing the anatomy of thalamo-cortical connectivity, explaining why thalamic sources favor inhibition, are given on lines 250 - 252.
2.2	"In addition, the authors should discuss whether the type of model they developed has enough biological detail to offer novel insights into mechanisms of brain rhythm generation and their modulation. Many detailed circuit models and modeling platforms are now available that have competing explanations for the origin of alpha (e.g. see eLife. 2020; 9: e51214.). Authors should compare their model against some of these other models/tools."	We reference some literature that discusses the variety of mechanisms of brain rhythm generation and modulation captured by our model. While we recognize that there are alternative modeling frameworks available, our focus in this study was on solving the inverse problem – inferring model parameters from fits to EEG data. Complex models necessarily involve a large number of unknown parameters. While they may be useful for exploring the forward problem they quickly become prohibitive when trying to solve the inverse problem. We believe our model provides a balance between having sufficient detail and being simple enough to invert.	References 19, 22 and 26 discuss the phenomena captured by our model. The challenges with solving the inverse problem with increasing model complexity are discussed in our earlier paper (reference 33).
2.3	"As far as organization of the manuscript, the authors should move the figures in Discussion into the Results, along with the description of those figures."	This problem was mostly due to the need for the figures to be on separate pages, which placed them after the results section even though they were referenced first in the results section.	We maintain the citation of figures 2, 3, and 4 in the results section and have moved the figures accordingly to strictly follow the submission guideline stating that figure captions are inserted immediately after the first paragraph in which the figure is cited. We believe that figure 5 is still better suited to be in the discussion section since it is a cross-check of the results.

No	Suggestion, question, or comment from Reviewer #2	Author's response	Change in the manuscript
2.4	Detailed comments:<br><br>"field models - external input to inhibitory neurons in cortex responsible for attenuating alpha"	It appears that some of the following comments are just notes, rather than questions. Nevertheless, we will try to answer them where possible.	
2.5	"they fit EEG data with eyes open (alpha higher) and closed (alpha lower) using population model and found that one parameter - external input to inhibitory neurons in cortex was responsible for modulating alpha power. that's not so surprising - but what is the explanation? does it fit the neuroanatomical data? mechanistic models?"	Our discovery that just a single parameter was responsible for alpha-blocking was surprising to us. Previous studies (references 31, 32) had instead found that multiple parameters were needed to explain alpha attenuation. Our neuroanatomical explanation, starting on line 246, is that the visual stimulus increases thalamo-cortical input to occipital cortex, increasing both p_ei and p_ee, but that the effect of p_ei dominates.	Our discussion about a neuroanatomical rationale is given the paragraph starting on line 246.
2.6	"why would opening eyes increase drive to cortical inhibitory neurons? which pathway is responsible?"	We speculate that thalamo-cortical signals to inhibitory cortical neurons are the dominant pathway by which a visual stimulus attenuates the alpha rhythm.	Our discussion about how opening of the eyes increases drive to inhibitory cortical neurons is given in the paragraph starting on line 246.
2.7	"there are many models that can account for the data ..."	We have described how model unidentifiability means that many of the details in a model cannot be learned by simply fitting to data, even if the fits are accurate. To address this problem we have outlined a method that finds the simplest explanation that still fits the data. We find that the result, in this case, does have a plausible neuroanatomical rationale.	

2.8	"105-108:<br><br>Local equations are linearized around a fixed point and the power spectral density (PSD) is derived assuming a stochastic driving signal of the excitatory population that represents thalamo-cortical and long range cortico-cortical inputs, assumed to be Gaussian white noise. The modelled PSD can then be written as a"<br><br>Why is thalamocortical drive assumed to be white noise? Is that realistic given knowledge of thalamocortical dynamics? I would think that some peaks in frequency, e.g. in alpha range would be more realistic.<br><br>OK, then later they mention that the inputs are not white noise, so that's a fittable parameter that influences the noise type provided (white, pink, brown, etc.)."	We emphasize that the alpha spectrum emerges from the dynamics of the cortical response, not from a specifically shaped input.	
2.9	"plos comp bio thalamic model - more realistic and offers more plausible insights into mechanisms of rhythm generation"	Again, there is a delicate balance between model complexity and invertibility. We find that our model, though simpler than others, is still extremely challenging to invert.	
2.10	"DJS - nice measure for quantifying differences in power spectra"		
2.11	"Fig.3 may have too much detail for the typical reader. Is there a way to summarize the fitted distributions for each patient rather than displaying 23 x 5 distributions??"	We wish to highlight the categories of parameters we use: some of them are forced to be same in eyes-open and eyes-closed states, while others are allowed to be different. It is unclear to us how to simplify this plot without compromising this objective.	

2.12	"Line 188-190: if most parameter responses are 0 or insignificant trend with degree of alpha blocking, why not instead show the parameter response that are significant or not 0??"	We examined trends in the differential response of 9 parameters and show how most exhibit little trend vs alpha-blocking. We believe it is important to show how most parameters have a weak response	
2.13	"The beginning of the Discussion and Figures 4 and 5 should be moved into the Results."	See response 2.3.	
2.14	"Although the discussion around lines 221 address some of this, can the authors comment on the mechanism as to why the parameter p_ei (excitatory input to inhibitory neurons) is the major determinant of changes in alpha between the EO and EC conditions and why p_ee is not important? I would have thought both parameters should influence the magnitude of oscillations. In addition, which neuroanatomical pathway would set the p_ei value and how would that pathway influence only the interneurons? Is the model-predicted parameter influencing alpha consistent with experimental data?"	Our explanation is that the visual stimulus increases the thalamo-cortical input to occipital cortex, probably increasing both p_ei and p_ee as suggested. Despite this, the effect of p_ei dominates because (i) the inhibitory neurons are more sensitive, as inferred in this paper and (ii) previous anatomical studies have shown that thalamo-cortical connectivity is greater to inhibitory neurons. Because we only see the effect on the cortex, we only infer the presence of p_ei, not p_ee.	We have added line 256 to explain why, even if p_ei and p_ee both increase together, it is still the inhibitory response that dominates the response of the cortex.
